# Effect of the COVID-19 pandemic on advanced life support units’ prehospital management of the stroke code in four Spanish regions: an observational study

**DOI:** 10.1186/s12873-023-00886-8

**Published:** 2023-10-04

**Authors:** Nicolás Riera-López, Francisco Aranda-Aguilar, Montse Gorchs-Molist, Jose Antonio Iglesias-Vázquez

**Affiliations:** 1 Servicio de Urgencias Médicas de Madrid (SUMMA-112), Madrid, Spain; 2 Córdoba Provincial Service. Centro de Emergencias Sanitarias 061 (CES 061), Córdoba, Spain; 3 Sistema d’Emergències Mèdiques (SEM), Barcelona, Spain; 4 Fundación Pública Urxencias Sanitarias de Galicia-061 (FPUSG-061), Santiago de Compostela, Spain

**Keywords:** Stroke, COVID-19, Emergency medicine, Emergency medical service, Stroke code, Neuroprotective

## Abstract

**Introduction:**

Stroke is the most common time-dependent pathology that pre-hospital emergency medical services (EMS) are confronted with. Prioritisation of ambulance dispatch, initial actions and early pre-notification have a major impact on mortality and disability. The COVID-19 pandemic has led to disruptions in the operation of EMS due to the implementation of self-protection measures and increased demand for care. It is crucial to evaluate what has happened to draw the necessary conclusions and propose changes to improve the system’s strength for the future. The study aims to compare prehospital time and neuroprotective care metrics for acute stroke patients during the first wave of COVID-19 and the same periods in the years before and after.

**Methods:**

Analytical, observational, multicentre study conducted in the autonomous communities of Andalusia, Catalonia, Galicia, and Madrid in the pre-COVID-19 (2019), “first wave” of COVID-19 (2020) and post-COVID-19 (2021) periods. Consecutive non-randomized sampling. Descriptive statistical analysis and hypothesis testing to compare the three time periods, with two by two post-hoc comparisons, and multivariate analysis.

**Results:**

A total of 1,709 patients were analysed. During 2020 there was a significant increase in attendance time of 1.8 min compared to 2019, which was not recovered in 2021. The time of symptom onset was recorded in 82.8% of cases, and 83.3% of patients were referred to specialized stroke centres. Neuroprotective measures (airway, blood glucose, temperature, and blood pressure) were performed in 43.6% of patients.

**Conclusion:**

During the first wave of COVID-19, the on-scene times of pre-hospital emergency teams increased while keeping the same levels of neuroprotection measures as in the previous and subsequent years. It shows the resilience of EMS under challenging circumstances such as those experienced during the pandemic.

## Introduction

Stroke is the second leading cause of overall mortality in Spain and the leading cause of disability in adults [1, 2]. One in six people will suffer a stroke in their lifetime [3]. The safety and effectiveness of treatments in ischemic stroke (fibrinolysis and mechanical thrombectomy) depend on the time that elapses from the onset of symptoms to reperfusion of the affected brain area. Emergency Medical Services (EMS) are critical in reducing this period [4–7].

In our country, health competencies are transferred to the seventeen Autonomous Communities (AACC). Since 1998, each of them has been implementing the procedure for action in patients with acute stroke (AS), called “stroke code” (SC) [8, 9]. As recommended by international guidelines, this protocol prioritizes transferring patients with symptoms compatible with AS to a hospital with a Stroke Unit (SU), pre-notifying the on-call neurologist [10–12].

EMS in Spain are highly professionalised. Although they are services that depend on regional governments, they are quite homogeneous in terms of how they operate and the protocols in place. They are among the few in the world to have medical and nursing staff in their advanced life support ambulances (ALSA). These units are responsible for most time-sensitive pathologies such as AS, acute myocardial infarction, cardiac arrest, or major trauma. They also deal with highly complex urgent medical pathologies. There are also basic life support ambulances (BLSA), which are more numerous and responsible for less complex emergencies.

The Health Emergency Centre of Andalusia (CES-061), the Medical Emergency System of Catalonia (SEM), the Medical Emergency Service of Madrid (SUMMA 112), and the Public Health Emergencies Foundation of Galicia-061 (FPUSG-061) are four important SEM in the country with responsibility for care in territories with very different rates and population distribution, demographic characteristics, climatological and resources. They serve 25.33 million inhabitants (8.43 million Andalusians, 7.56 million Catalans, 6.64 million Madrilenians, and 2.7 million Galicians), being 53.96% of the Spanish population in 2021 [13].

The infection with the novel coronavirus (SARS-COV-2) had an unprecedented impact on the world’s population. As of September 2021, more than 225 million people suffered from the disease due to the new coronavirus (COVID-19), with more than 4 million deaths [14]. Spain was one of the countries that suffered the most from the impact of the pandemic. Until September 2021, almost 5 million infected and more than 82,000 deaths were recorded [15], assuming the first cause of mortality in 2020 [16].

The pandemic put the health system in unprecedented difficulties. There is evidence of the negative impact it had on time-dependent pathologies, such as the observed reduction in admissions for acute coronary syndrome or AS in hospital centres during the first wave of COVID-19 [17–22]. However, few analyses have focused on the behaviour of EMS in managing CS during this pandemic.

In line with the findings of international institutions and scientific societies, it is necessary to evaluate what has happened so far to draw the necessary conclusions and propose changes that will improve the system’s strength for the future [23]. The main aim of this study was to compare the time spent caring for patients with AS. And the secondary aim is to analyse the differences in the management of SC that have been attended during the first wave of the COVID-19 pandemic, compared to the same periods of the previous and subsequent years.

## Materials and methods

An analytical, observational, and multicentre study of patients treated with suspected AS by EMS in the AACC of Andalusia, Catalonia, Galicia, and Madrid was conducted.

Three time periods were investigated: from March 1 to May 31, 2019 (pre-COVID-19), from March 1 to May 31, 2020 (first wave of COVID-19), and from March 1 to May 31, 2021 (post-COVID-19).

Inclusion criteria: all records of patients with SC activation cared for by an ALSA with a physician onboard and with data for at least one recorded vital constant. Exclusion criteria: records of patients being transferred between two hospitals, those attended only by BLSA without a doctor, and in which the times that constitute the study’s main aim were not recorded.

Non-randomized consecutive sampling was used. Data were collected manually from the corresponding patient follow-up software applications in each of the EMS and securely recorded in the European RES-Q registry [24], where statistical analysis was performed.

### Variables

Sociodemographic and generic: Record the patient’s filiation (Yes/No). Age (years). Gender (male/female). Municipality with an interventional hospital (Yes/No). Type of resource served: ALSA/BLSA/Air ambulance.

**Fig. 1:**
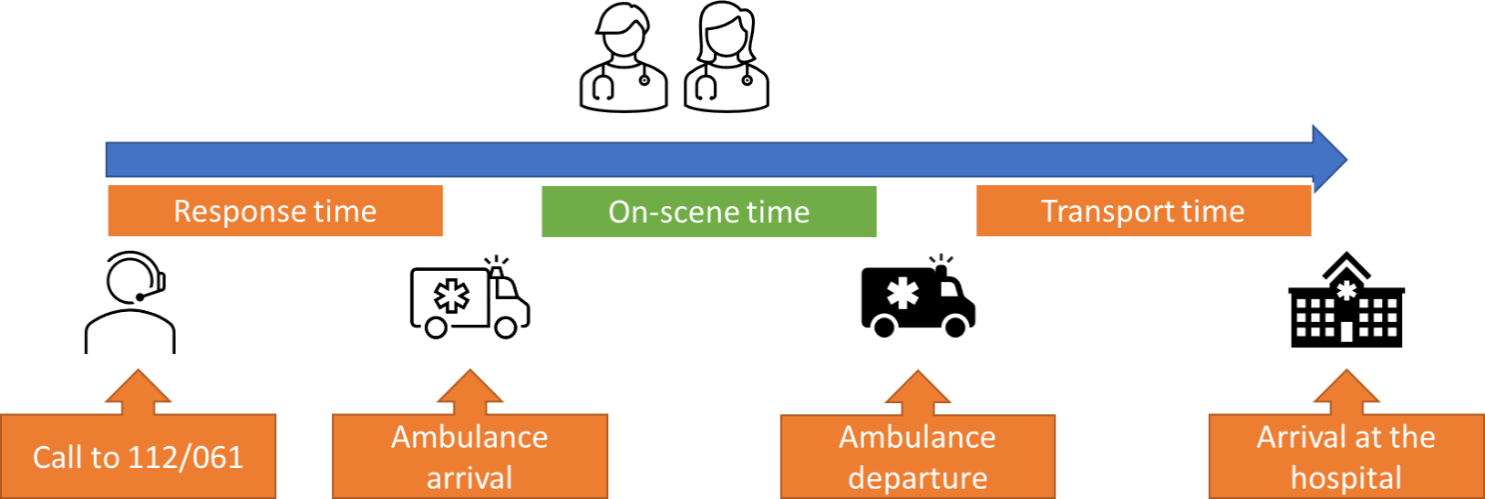
Diagram of the periods collected

For the main aim, the following periods were collected (Fig. 1): Ambulance response time (ART): time from when the coordination centre activates a resource until the care unit arrives at the place where the patient is. Assistance time or on-scene time (OST): Time from when the care unit starts the assistance at the events scene until the start of the transport to the hospital centre. Ambulance transportation time (ATT): transfer time to the hospital.

For the secondary aim, the following variables:

Vital signs: Systolic blood pressure (SBP) and diastolic blood pressure (DBP), (mmHg), heart rate (beats/min), respiratory rate (breaths/min), peripheral oxygen saturation (%), Glasgow coma scale (GCS, 3–15), glycemia (mg/dl), temperature (°C).

CI Process: Is the onset time of symptoms collected? (Yes/No). Anticoagulated patient (Yes/No). History of atrial fibrillation (AF) or de novo AF (Yes/No). Destination hospital level for treating patients with AS: Primary centre, where the patient can access a computerized tomography (CT scan) and fibrinolytic treatment. Specialized centre: one in which the patient can access thrombectomy and ED. Any other centre.

Neuroprotection: Actions on airway-intubation (Yes/No). Actions to correct glycemia (Yes/No). Actions to correct the temperature (Yes/No). Actions to correct blood pressure (Yes/No). Actions to correct saturation-oxygen therapy (Yes / No).

### Statistical analysis

Registries with missing data on ART, OST and ATT were excluded. Missing values for the secondary outcome variables were addressed by excluding cases with missing data from the analysis. This approach resulted in a reduction in sample size for some of them. Outliers were identified using box plots and removed after visual inspection (calculated as the upper and lower 5% quantile of each of the three periods).

The results are expressed in means, standard deviations, or median and interquartile range for quantitative variables, absolute frequencies, and percentages for qualitative variables. Neuroprotection activities were reported on the total number of patients and their fraction with altered clinical findings. Thus, it was considered necessary to act on the airway when the GCS was less than 9, on glycemia when it was higher than 180 mg/dl, on temperature when it was equal to or greater than 37.5º, on the blood pressure when it was higher than 220/185, and finally on oxygen saturation when it was less than 94% [10].

An inferential analysis compared the means using ANOVA, or Kruskal Wallis, using Tukey’s correction for post hoc testing. The proportions were compared by Fisher’s exact test or Chi-square, as appropriate. A p-value < 0.05 was considered statistically significant, calculating 95% confidence intervals.

Data were analysed with RStudio statistical software (RStudio®, PBC, Boston, MA).

All data was processed following the European Data Protection Regulation 2016/679. The Research Ethics Committee of Santiago-Lugo, at its meeting on May 23, 2021, issued a favourable opinion of the study. The confidentiality of the subjects included in the study was always guaranteed, both in the storage and the presentation of results under the Organic Law on the Protection of Personal Data (Organic Law 3/2018, of December 5, Protection of Personal Data and guarantee of digital rights). The data used in this study is available upon a reasonable request to the corresponding author, and after permission of all participating services.

## Results

**Fig. 2:**
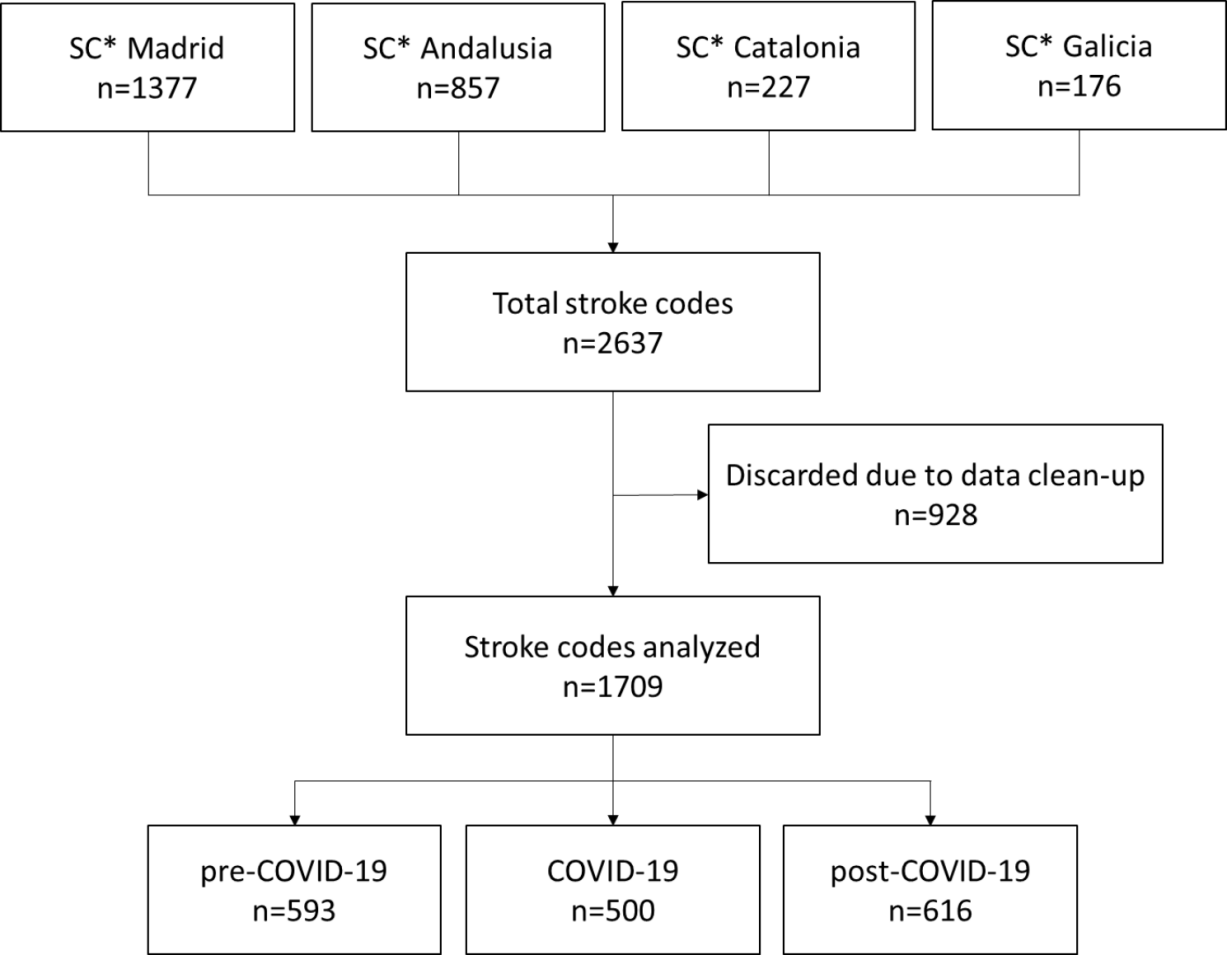
Flowchart with the stroke codes analysed * SC: Stroke codes transferred by advanced life support ambulance.

1,709 patients attended by ALSA units with activation of the SC protocol in the four AACC during the temporary study period (March, April, and May 2019, 2020, and 2021) were analysed (flow diagram as in Fig. 2). Baseline characteristics are shown in Table 1. During the first wave of COVID-19, 15% fewer patients were treated than the previous year, and 18% fewer patients than in the year after. No significant differences in vital signs were found between the three periods. The median age was 74 years (63–82), significantly younger during the first wave of the pandemic than during 2019 (-2.08; CI: -4.07;-0.09) and 2021 (-3.74; CI: -1.76;-5.71). The proportion of women was 46.5%. The characteristics of the population are analysed in Table 2.

**Table 1:** Baseline characteristics

	pre-COVID-19	COVID-19	post-COVID-19	p-value	Test	M
	n = 593	n = 500	n = 616			
Age years (median [IQR])	74.00 [63.00, 82.00]	72.00 [60.00, 80.00]	75.00 [65.00, 83.00]	< 0.001*	3	
Sex = Female (%)	276 (46.5)	228 (45.6)	290 (47.1)	0.886	1	
Ambulance response time minutes (median [IQR])	10.98 [7.97, 14.42]	11.00 [8.10, 15.00]	11.11 [8.00, 15.05]	0.248	3	
On-scene time minutes (median [IQR])	30.28 [24.23, 37.98]	33.00 [24.51, 41.87]	32.00 [24.84, 39.35]	0.025*	3	
Ambulance transport time minutes (median [IQR])	13.03 [8.10, 21.00]	12.77 [8.07, 19.69]	13.76 [9.00, 23.00]	0.033*	3	
Collects onset time = Yes (%)	482 (81.3)	424 (84.8)	509 (82.6)	0.304	1	
Patient affiliation = Yes (%)	531 (89.5)	431 (86.2)	591 (95.9)	< 0.001*	1	
Background = Yes (%)	128 (21.6)	81 (16.2)	140 (22.7)	0.017*	1	
Anticoagulation = Yes (%)	88 (14.8)	74 (14.8)	87 (14.1)	0.921	1	
Blood glucose mg/dl (mean (SD))	137.28 (54.13)	140.63 (65.54)	132.88 (57.58)	0.088		2
Heart rate bpm (mean (SD))	83.29 (22.34)	83.27 (22.05)	82.16 (23.26)	0.617		3
Respiratory rate rpm (median [IQR])	14.00 [14.00, 16.00]	14.00 [14.00, 16.00]	14.00 [14.00, 16.00]	0.053	3	38
Systolic blood pressure mmHg (mean (SD))	157.67 (30.65)	155.10 (31.48)	155.62 (29.74)	0.328		2
Diastolic blood pressure mmHg (mean (SD))	88.56 (20.47)	87.95 (21.00)	86.37 (19.66)	0.159		10
Oxygen saturation % (median [IQR])	96.00 [94.00, 98.00]	96.00 [95.00, 98.00]	96.00 [95.00, 98.00]	0.208	3	7
Temperature ºC (mean (SD))	35.95 (0.59)	35.97 (0.73)	36.00 (0.58)	0.514		99
Glasgow Coma Scale (median [IQR])	15.00 [12.00,15.00]	15.00 [12.00,15.00]	15.00 [12.00,15.00]	0.413	3	6
**Glasgow Coma Scale (%)**				**0.916**		**6**
3–8	49 (8.3)	37 (7.4)	43 (7.0)			
9–12	127 (21.6)	113 (22.7)	135 (21.9)			
13–15	413 (70.1)	348 (69.9)	438 (71.1)			
**Region (%)**				0.088		
Andalusia	170 (28.7)	153 (30.6)	179 (29.1)			
Barcelona	57 ( 9.6)	66 (13.2)	50 ( 8.1)			
Galicia	41 ( 6.9)	34 ( 6.8)	53 ( 8.6)			
Madrid	325 (54.8)	247 (49.4)	334 (54.2)			
Municipality with hospital = Yes (%)	409 (69.0)	331 (66.2)	394 (64.0)	0.182	1	
**Destination hospital level (%)**				**0.769**		
Specialized	488 (82.3)	416 (83.2)	520 (84.4)			
Primary	104 (17.5)	82 (16.4)	95 (15.4)			
Other	1 (0.2)	2 (0.4)	1 (0.2)			
**Type of transport (%)**				0.393		
Air ambulance	6 (1.0)	10 (2.0)	10 (1.6)			
ALSA	587 (99.0)	490 (98.0)	606 (98.4)			
**Neuroprotection measures**						
Airway action = Yes (%)	34 (5.7)	14 (2.8)	24 (3.9)	0.054	1	
Oxygen saturation = Yes (%)	104 (17.5)	89 (17.8)	104 (16.9)	0.913	1	
Temperature = Yes (%)	13 (2.2)	11 (2.2)	11 (1.8)	0.846	1	
Blood glucose = Yes (%)	47 (7.9)	26 (5.2)	42 (6.8)	0.198	1	
Blood pressure = Yes (%)	62 (10.5)	44 (8.8)	60 (9.7)	0.660	1	

SD: standard deviation. IQR: interquartile range. Test: No specification: Chi-square (qualitative) or Student’s t (quantitative). (1) Fisher’s exact test. (2) Kruskal Wallis. * p < 0.05. M: missing.

**Table 2:** Characteristics of the population analysed

	Andalusia	Catalonia	Galicia	Madrid
Population	8.538.376	7.792.611	2.690.464	6.750.336
Density (inhabitants/km^2^)	97	239	91	841
Provinces	8	4	4	1
Stroke Centres	5	6	3	11
Air ambulances	5	4	2	2
ALSA	30	65	12	27
Calls received 2020	3.585.146	3.198.252	1.280.305	1.456.526
Resource mobilization 2020	826.626	138.111	240.636	291.651
Inhabitants/Stroke Centre	1.707.675	1.298.769	896.821	613.667

**Fig. 3:**
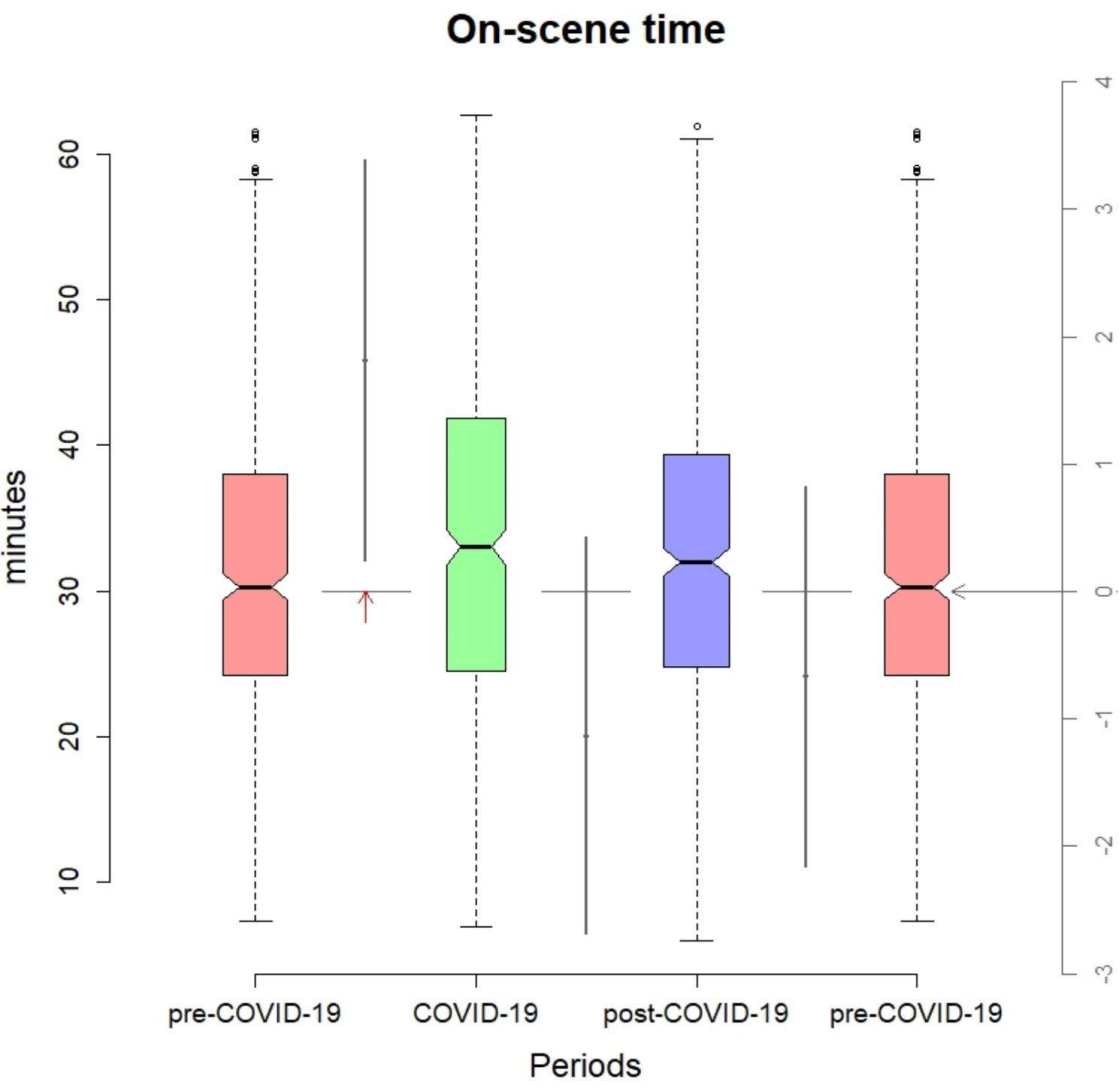
Box plot of “On-scene time” and 95% confidence intervals of the differences Red arrow shows significance of pairwise differences (p = 0,025).

Analysis of the periods spent showed a median ART of 11 min (8-14.9), an OST of 31.6 min (24.5–39.8), and an ATT of 13.1 min (8.35-21). During 2020 there was a significant increase in OST (33.2; CI95%: 32.2–34.3) of just 1.8 min compared to 2019 (31.4; CI95%:30.6–32.3). There are no significant differences between the pre-COVID-19 and post-COVID-19 period (Fig. 3). The rest of the periods were either unaffected or reduced during 2020.

Regarding the SC protocol, in 82.8% of cases, the time of onset of symptoms was recorded. 83.3% of patients were referred to specialized stroke centres, with 16.4% of patients going to primary stroke centres and around 0.2% to other centres.

**Fig. 4:**
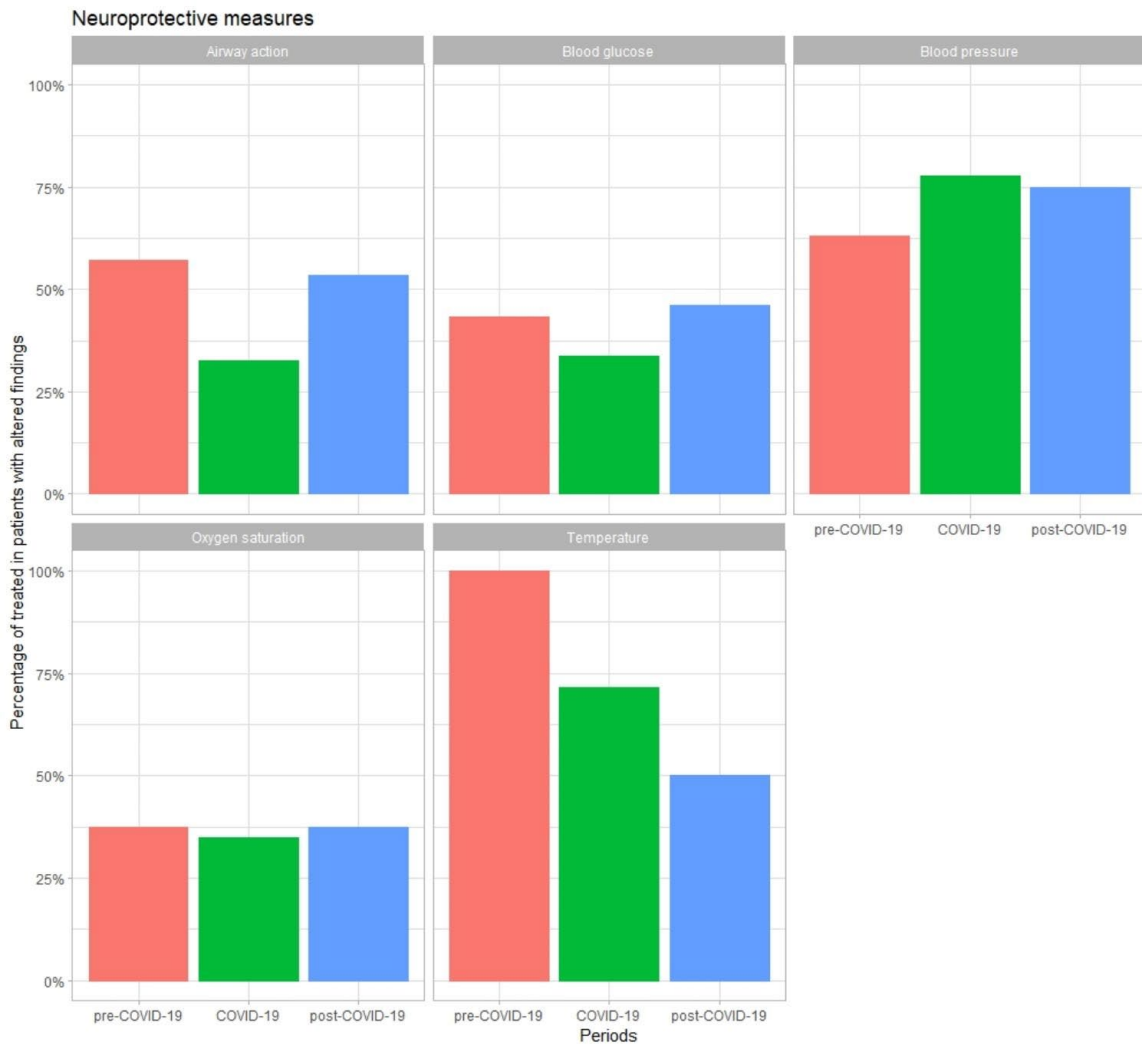
Proportion of neuroprotective measures on patients with pathologic vital signs

Pathological findings in vital signs were found in 554 patients (32.5%). In 305 (43,6% of those with pathological findings) neuroprotection measures (airway, blood glucose, temperature, or blood pressure) were performed (Fig. 4). In 63 (48.8%) patients with GCS less than 9, airway isolation was performed. During 2020, the proportion was reduced non-significantly to 32.4% compared to 57.1% in 2019 and 53.5% in 2021. Oxygen therapy was administered in 103 (36.7%) patients with hypoxemia. Antipyretic treatment was instituted in 8 (66.7%) patients with fever. In 93 (41.3%) patients with hyperglycaemia, treatment was administered to reduce it. In 38 (71.7%) patients with systolic hypertension, treatment was instituted to normalize it. None of the above measures had statistically significant differences between the three periods.

The bivariate analysis showed a weak correlation between attendance time and the following parameters: low saturation, low GCS, and high glycemia (r = 0.1, p < 0.05).

## Discussion

It is the first study to analyse SC treated in four AACC in the prehospital setting during the COVID-19 pandemic. Although OST were more prolonged than recommended by international guidelines, they barely lasted during the pandemic (8.9% increase 2019–2020: 1.81 min; CI95%: 0.24 to 3.38), recovering during the post-COVID-19 period at the same levels as in the pre-COVID-19 period. The application of neuroprotection measures did not vary significantly.

The findings on the reduction in SC during the pandemic, and the decrease in the median age of patients seen, are consistent with the findings of other studies [17, 18, 25]. The main hypotheses cited are the fear of being infected in hospitals, especially in the older population.

Regarding the main aim, 25% of the ART and 10% of the OST in our series met the American Stroke Association targets [12]. During the first wave of the pandemic, OST increased about 10%. The largest study analysing prehospital times, conducted in the United States with more than 180,000 patients, concluded that 76% of ART and 46% of OST patients met the target [26]. In a Busan study on the southeast coast of Korea, OSTs, which were almost 66% lower than those in our series, were prolonged during the pandemic by about 30% [27]. In another study in Bangkok, however, ARTs increased 5-fold and OSTs nearly 2-fold during COVID-19 [28]. The security measures against the new coronavirus, specifically the process of placing Personal Protective Equipment, can explain the prolonged OST of the works analysed. An explanation can also be found for the fact that ALSA units have a longer time on stage than BLSA units (e.g., the case of Busan), since they can stabilize the patient and perform different medical actions such as the neuroprotection measures discussed below. Within the studies analysed, our series is one of those that registered the smallest increases in time.

The vital signs recorded showed the same patterns as those published in other articles [29, 30]. There were no significant differences during the first wave of the pandemic except for temperature, which was clinically relevant to this study. The recording of the onset of symptoms in 85% of cases is in line with that published by other groups (75-97%) [31, 32]. The decision to indicate reperfusion treatment is based on this data so that it can be improved. On the other hand, we have not found articles that analyse the percentage of patients with a record of personal history or anticoagulant treatment at the prehospital level.

Teams specialized in advanced life support with a doctor on board can establish corrective measures in the prehospital setting that improve the prognosis of patients with AS (e.g., reducing excessively high blood pressure levels, correcting hypoxia, and alterations in glycemia or temperature) [33]. The figures above 80% of assessment records and 43.6% of effective neuroprotection measures are positive but can be improved. Few studies are looking at such measures. In a Rochester, New York study, capillary glycemia was performed in 84% of patients, a 12-lead electrocardiogram in 67%, and vascular access in 73% of patients [34]. Although not all manoeuvres are recommended with a high level of evidence by international guidelines on the prehospital management of AS [10, 12, 35], all of them are included in the protocols of each AACC and endorsed by the corresponding scientific committees. One of the most controversial measures is the control of blood pressure in the pre-hospital setting. However, it is a criterion for access to fibrinolytic treatment in the acute phase, and although decreases must be progressive over 24 h, it is an opportunity for the patient to receive one of the reperfusion therapies.

Finally, in the multivariate analysis, patients with more significant complications (low GCS, low saturation, airway management, or need to correct glycemia) showed a weak correlation with increased OST. There is no evidence on whether the neuroprotection measures practiced in ALSA in critically ill patients, with the consequent increase in care times, condition a worse prognosis compared to delaying these measures to the hospital setting, prioritizing drastically shortening care times. However, without more comprehensive studies, selected patients should be treated by ALSA.

Limitations.

Firstly, being an observational study, selection bias was mitigated by consecutive sampling of all cases. In addition, 35% of records were dropped due to the data cleaning process, a method commonly used in research studies to drop outliers that may bias the results. Secondly, it was limited to the pre-hospital setting, so only cases that activated EMS were considered. These tend to be the most severe patients who call 112/061, choosing not to go to hospital by their own means. Finally, only patients with a pre-hospital diagnosis of suspected SA were included, but there are no data on the confirmation of the final hospital diagnosis. Nor was it possible to obtain the information on the outcome of these patients (such as mRS at 30–60 days) that is usual in these types of studies. Future studies should include in-hospital clinical and outcome variables (Table 3).

**Table 3:** Strengths and limitations of the study

Strengths	Limitations
**First analysis of its kind.** It is the first to analyze stroke care in four Spanish regions in the prehospital setting during the COVID-19 pandemic.	**Observational study.** Possibility of selection bias as only cases that activated EMS were considered, and there is no information on the confirmation of the final hospital diagnosis.
**Consistency with other studies.** The findings of the study are consistent with previous research.	**Limited data on outcomes.** It lacks information on the outcome of the patients, such as the modified Rankin Scale (mRS) at 30–60 days, which is a common measure in stroke studies.
**Comparison with previous studies.** The study compares its results with other studies conducted in different regions and settings, providing a broader context and allowing for a better understanding of the findings.	**Data cleaning process.** The study dropped 35% of records due to the data cleaning process, which may introduce another source of bias.
**Analysis of neuroprotection measures.** The study examines the application of neuroprotection measures in the prehospital setting during the pandemic and reports that there were no significant changes in their implementation.	**Limited generalizability.** The study focuses on four specific Spanish regions during the COVID-19 pandemic, which may limit the generalizability of the findings to other settings or regions.
**Resilience of EMS.** Despite the challenges posed by the pandemic, the EMS system was able to recover and provide care at pre-pandemic levels during the post-COVID-19 period. This finding emphasizes the effectiveness and adaptability of EMS in such crises.	

## Conclusion

During the first wave of COVID-19, the number of SC attended was reduced by more than 10%, and assistance times increased, but the application of neuroprotection measures did not suffer significant changes. The analysis of the post-pandemic period has revealed a recovery of metrics to pre-pandemic levels and an increase in SC above pre-pandemic levels. It shows the remarkable resilience of EMS in situations as complex as the pandemic experienced in 2020. Further studies are needed to elucidate whether the time spent on neuroprotection measures at the prehospital level influences the prognosis of patients with AS.

## Data Availability

The anonymized data used in this study is available upon a reasonable request to the corresponding author, and after permission of all participating services.

## Abbreviations

AACC: Autonomous communities
AF: Atrial fibrillation
ALSA: Advanced life support ambulance
ART: Ambulance response time
AS: Acute stroke
ATT: Ambulance transportation time
BLSA: Basic life support ambulance
CES-061: Centro de Emergencias Sanitarias de Andalucia 061
COVID-19: Coronavirus disease 2019
CT: Computed tomography
DBP: Diastolic blood pressure
EMS: Emergency Medical Service
FPUSG-061: Fundación Pública Urxencias Sanitarias de Galicia-061
GCS: Glasgow coma scale
OST: On-scene time (time spent in face-to-face patient care)
SARS-COV-2: Severe acute respiratory syndrome coronavirus 2
SBP: Systolic blood pressure
SC: Stroke code
SEM: Sistema de Emergencias Médicas de Cataluña
SU: Stroke Unit
SUMMA-112: Servicio de Urgencias Médicas de Madrid 112
